# Jumping the gun: Faster response latencies to deceptive questions in a realistic scenario

**DOI:** 10.3758/s13423-016-1218-z

**Published:** 2017-03-13

**Authors:** Tessa Mapala, Lara Warmelink, Sally A. Linkenauger

**Affiliations:** Department of Psychology, Lancaster University, Lancaster, LA1 4YF UK

**Keywords:** Human factors, Judgment and decision making, Social cognition, Attention, Executive control

## Abstract

Most theories of lie detection assume that lying increases cognitive load, resulting in longer response latencies during questioning. However, the studies supporting this theory are typically laboratory-based, in settings with no specific validity in security contexts. Consequently, using virtual reality (VR), we investigated how response latencies were influenced in an ecologically valid environment of interest to security professionals. In a highly realistic airport security terminal presented in VR, a security officer asked participants yes/no questions about their belongings. We found that liars actually responded more quickly to questions on which they were lying than to questions on which they were telling the truth. A control group, who answered the same questions but were not lying, answered equally quickly for all questions. We argue that this decrease in response time is possibly an unconscious reaction to questions on which individuals must answer deceptively. These results call into question the generalizability of previous research and highlight the importance of ecological validity when researching lie detection. These findings also uncover a new potential tool for enhancing lie detection in real-world scenarios.

Due to increases in the number of terrorist attacks internationally this century (Global Terrorism Database, 2016), security concerns have become more prevalent. Events and venues that attract large crowds are targeted to inflict maximum damage (Morris, 2015). Security checkpoints act as the main bastion of protection at these events (e.g., at stadiums and transport hubs), and security personnel are specifically tasked with detecting those with hostile intent trying to pass through security (e.g., smuggling weapons). However, people, even police officers and other experts, are quite poor at detecting deception, with a bias toward assuming that people are telling the truth (Bond & DePaulo, 2006). Different physiological lie detectors have been tested as compensatory methods, but they have not been found sufficiently reliable (Vrij, 2008), nor would they be able to deal with the large numbers of people passing through checkpoints.

Response latencies, the time between the end of the question and the start of the answer, have been considered a promising alternative, because this is one of the more consistent vocal cues to deception (Vrij, 2008). Response latency is considered a measure of cognitive load, both within the lie detection literature (e.g., Vrij, Edward, & Bull, 2001) and in other psychological fields (e.g., research on driving by Lamble, Kauranen, Laakso, & Summala, 1999). Cognitive load can also be measured by means of a secondary task. Several forms of the detection response task have been used—for example, during driving simulations (Bruyas & Dumont, 2013)—and a similar tactile task has been used for lie detection in a laboratory setting (Lancaster, Vrij, Hope, & Waller, 2013). However, secondary tasks have not yet been used in lie detection studies that aim for higher ecological validity, since these tasks are not in general use in applied settings. Response latency is a cognitive-load cue that is automatically present in any interview, and therefore is more attractive for use in real life.

Deception creates cognitive load, since individuals must inhibit their true memories while simultaneously fabricating a fictitious account. This increased load in liars then causes them to respond more slowly to questions. However, several other theories about deception also make predictions about response latencies (Sporer & Schwandt, 2006). The attempted-control theory, which holds that liars try to imitate truth tellers as much as they can, suggest that liars could show shorter latencies. If liars believe that long latencies are a cue to deception, they may try to deliberately answer as quickly as possible. This hypothesis assumes that they are capable of doing so while also handling high cognitive load (Sporer & Schwandt, 2006). However, little if any support for this hypothesis has been found.

Sporer and Schwandt’s (2006) meta-analysis of paraverbal cues to deception showed that liars indeed showed longer response latencies. However, the effect size was relatively small (*d* = 0.18, *p* = .004), and the effect sizes were very heterogeneous across studies. The response latency effect was also affected by several moderators. The differences between truth tellers and liars were larger when people were discussing their feelings and facts than when they were only discussing facts. Giving participants time to prepare their statement reduced the difference between truth tellers and liars to nonsignificance, whereas motivating participants to do well increased it. The effect of response latency was larger in studies using within-subjects designs than in studies using between-subjects designs. Sporer and Schwandt concluded that the meta-analysis of response latencies supported the cognitive-load model and not the attempted-control model. DePaulo et al.’s (2003) meta-analysis showed a smaller effect size for response latency (*d* = 0.02), but agreed on the high heterogeneousness of the studies in this area. Like Sporer and Schwandt, they found that the difference between truth tellers’ and liars’ response latencies was affected by preparation. In unplanned deception tasks, liars’ latencies tended to be longer, but for planned deception, this effect was reduced.

These findings are reflected in people’s beliefs about latency as a deception cue. Hartwig and Bond’s (2011) meta-analysis showed that people associate an increase in latency with deception (*r* = .18). Colwell et al. (2006) studied US police officers and found that 65% of them believed that response latencies increase in liars telling spontaneous lies, 26% believed that liars had shorter latencies, and 14% believed there was no difference. Colwell et al. also found that, for planned lies, police officers were more divided: 38% believed liars had shorter latencies, whereas 37% believed liars had longer latencies, and 24% said there was no difference.

However, the situations in which we are interested in identifying deception differ drastically from the artificial, laboratory-based situations that were used in most studies in these meta-analyses. In such studies, participants are often asked to lie about arbitrary stimuli presented on a desktop display (e.g., Sheridan & Flowers, 2010; Vendemia, Buzan, & Green, 2005) or in response to open-ended questions from an innocuous research assistant (e.g., Walczyk, Mahoney, Doverspike, & Griffith-Ross, 2009). At security checkpoints, however, security personnel typically ask questions that require short answers or a yes/no response—for example, “Did you pack your own bag?” (Transport Security Administration, 2016). Unlike in laboratory studies, the questions usually are personally relevant to the responder in that particular situation. In these situations, both the questioner and the responder know the “right answer,” since the responder is aware that admitting to illegal acts is likely to lead to delay or arrest. Cognitive load may be lower in such a situation, since there is no need for the participant to actively remember their answers, which is necessary to maintain consistency between the answers in open-ended interview questions. This is one of the reasons that lying is considered more cognitively loading than truth telling (Vrij, Fisher, Mann, & Leal, 2006). At the same time, this paradigm requires speech and social contact, in a way that computer tasks do not. This could mean that cognitive load is increased when compared to laboratory tasks, because the participant is likely to monitor and attempt to control their behaviors and expressions to look truthful (Vrij, Fisher, Mann, & Leal, 2006). This is not necessary when completing a computer task. It is also likely that arousal is increased in a task that includes a social interaction, as compared to a computer task (Rauch, Strobel, Bella, Odachowski, & Bloom, 2014).

With recent advances in virtual reality (VR) technology, we can now create realistic, immersive environments that individuals experience as similar to their counterparts in the real world, even in forensic settings (Benbouriche, Nolet, Trottier, & Renaud, 2014; Mertens & Allen, 2007), and that are far superior to desktop displays (Sanchez-Vives & Slater, 2005). Using VR, we conducted an experiment to assess whether the findings of increased deceptive response latencies generalize to questions posed by a security checkpoint officer in a realistic virtual environment (VE)—specifically, an airport security checkpoint. If the cognitive-load theory generalizes to these real-world scenarios, we should expect liars to take longer to respond. However, if these types of real-world scenarios reduce cognitive load, other factors, such as arousal or attempted control, may lead to no difference, or even to faster response times for liars than for truth tellers.

## Method

### Participants

Forty undergraduate students from Lancaster University were recruited to take part in this study, using opportunity sampling and the Lancaster University participant pool (32 female, eight male; *M*_age_ = 19.18, *SD*_age_ = 2.76). All participants were native or fluent English speakers. All had normal or corrected-to-normal vision to participate in the VR scenario, and all provided informed consent.

### Design

For the study we used a 2 (Deception) by 2 (Question Type) by 8 (Question) mixed design. The deception variable was varied between subjects: Half of the participants’ bags contained nonrestricted as well as restricted items, which they were instructed to lie about (deception condition). The other participants’ bags contained all nonrestricted items (nondeception condition). The questions type variable was varied within subjects: Half of the questions required those in the deception condition to lie (experimental questions), but they responded truthfully to the others (control questions). In the nondeception condition, participants responded truthfully to all 16 questions. Question was also a within-subjects variable: All participants were asked eight questions per question type. The dependent measure was the response latency—the time between the end of the question and the start of the answer. Responses were recorded using Audacity software, which allowed for millisecond precision. We also assessed immersion using a standard immersion questionnaire (Slater, Usoh, & Steed, 1994).

### Apparatus and stimuli

The study was conducted in a quiet room. The laptop running the VR program was placed on a table, enabling the oculus external tracker to be at eye level when participants were seated. The head-mounted display (HMD) used was the Oculus DK2, which provided both position and orientation tracking of head movements.

#### Backpack

An empty backpack was provided for participants to pack specific items, depending on their assigned condition. These items included the items prohibited through airport security—a large bottle of liquid, a hammer, scissors, and lighter fluid—as well as the control items—a chocolate bar, a pair of glasses in their case, a box of paracetamol, a t-shirt, and a notebook. This aspect of the experiment, packing a bag with items about which the deception questions were asked, was adapted from Mullins et al. (2014).

#### VR airport

A 3-D model of an empty industrial building was used to simulate the airport security VE, using the Unity3D software program. Objects typically found at airport security checkpoints, such as a metal detector and baggage X-ray machine, were put into this empty room. A stationary, armed airport security guard avatar was also present in the airport environment. The security guard avatar had his mouth covered throughout, so as to avoid issues with mouth movements not properly corresponding with the questions being asked. The security guard was also carrying a rifle, in an attempt to counteract the difficulty of mimicking the imposing atmosphere of real airport security checkpoints in this VR scenario. The VE was displayed through an HMD (Oculus DK2), which also had tracking technology enabled, allowing for participants’ head movements to correspond with visual changes in the VE.

#### Interview

Using text-to-speech technology, a set of 16 questions, comprising eight control questions and eight experimental questions (see Table 1), were programmed to emanate from the security guard avatar. The control questions elicited a truthful response from all participants, whereas the experimental questions elicited a deceptive response from those in the deception condition and a truthful response from the controls. The response latencies to the 16 interview questions were measured in milliseconds, from when the question had been uttered in full by the security avatar in VR to the moment that the participant began to respond.

**Table 1:** Interview questions: Control (C) and experimental/deception (E)

Question Type	Question
C	Are you currently a student?
C	Did you pack your bag yourself?
C	Are you carrying any food items?
C	Does your bag contain any electrical equipment?
C	Are you travelling on your own?
C	Do you have any explosives in your bag?
C	Are you carrying any tobacco products?
C	Are we currently in an airport?
E	Does your bag contain any prohibited items?
E	Does your bag contain any large quantities of liquids?
E	Are all the items in your bag within the travel guidelines?
E	Do you have any lighters or flammable liquids in your possession?
E	Are you sure there are no restricted items in your bag?
E	Are you carrying any sharp objects?
E	Are you carrying any items which could be used to harm someone?
E	Are you in possession of any tools or other dangerous items?

These questions were adapted from those in Mullins et al. (2014), with the aim of mimicking questions that might be realistically asked in an airport scenario. The number of questions was increased to 16, in order to obtain more robust data. Increasing the number of questions further would not be doable in an interview setting, where normally relatively few questions are asked; for example, the cognitive interview uses only four main questions, with the possibility of elaboration (Memon, Meissner, & Fraser, 2010). A second laptop was used to capture participants’ verbal responses, using the audio software program Audacity.

#### Immersion questionnaire

A presence questionnaire (see Appendix A) requiring responses to participants’ experience in the airport environment was administered. Six of the seven questions used a 7-point Likert scale, whereas the final, open-ended question required a general response that (1) helped enhance the realistic nature of the airport environment for future experiments, but (2) reduced the realism of the scenario. The open-ended question was merely used to gauge participants’ experiences, to improve the scenario in the future. The answers from this question were not included in the immersion analysis. The overall scores, which were calculated from the sum of the six Likert responses, were used to measure participants’ immersion in the VR environment.

This questionnaire was adapted from a similar immersion questionnaire developed by Slater, Usoh, and Steed (1994), to see whether there was a difference in the level of immersion, depending on condition.

### Procedure

**Fig. 1:**
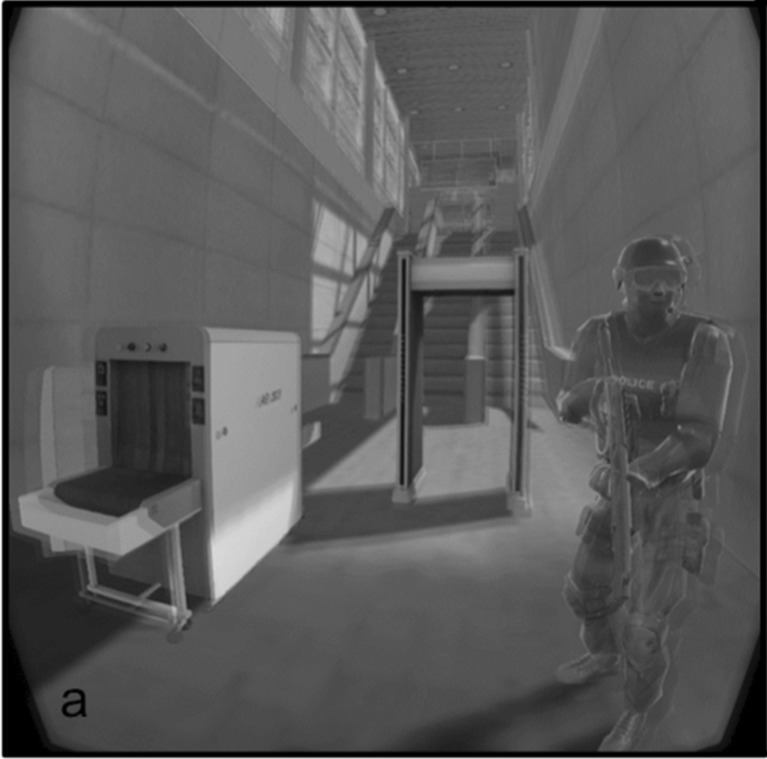
A screenshot of the virtual environment used in the study

After going through the informed-consent procedure, participants packed the bag with the items they were to take through the VE. Participants then donned the HMD and were immersed in a VE resembling an airport security checkpoint, complete with metal detectors and baggage scanners (see Fig. 1). Each participant was tested individually. They were first given a written brief, depending on their condition, informing them of the items that they would be given to pack for their airplane journey. Following this, the participants were presented with the items that they were to pack, and were given the same instructions verbally to explicitly specify which of the items were prohibited and which were not (if applicable), to ensure that they understood their task. Following this, participants then packed the backpack with these items (see Table 2). The HMD was then placed on the participant’s head, and he or she was immersed in the airport VE (see Fig. 1). Participants were then asked a set of 16 questions, in random order, while they were in VR and situated directly in front of the security guard avatar, and they gave verbal “yes” or “no” responses to each individual question. After answering all of the questions, the participants exited the VE and filled in the immersion questionnaire.

**Table 2:** Items packed by the control and deception groups

	Group	
	Control	Deception
Group-specific items	Box of painkillers	Large bottle of liquid
	T-shirt	Hammer
	Notebook	Flammable liquid
	Pair of gloves	Scissors
Shared items (control and deception groups)	Pair of glasses Chocolate bar	

## Results

Trials on which participants did not respond, answered the question incorrectly, or did not hear/understand the question were removed from the analysis, which accounted for 2.6% of the data. One participant’s data were removed due to immersion scores that were –4 *SD*s from the mean. Another participant’s data were not included due to a technical error with recovering the recorded data; thus, their immersion responses were not used. Immersion scores were quite high, *M* = 26.87, *SD* = 5.58, and did not differ between conditions, *p* = .410.

**Fig. 2:**
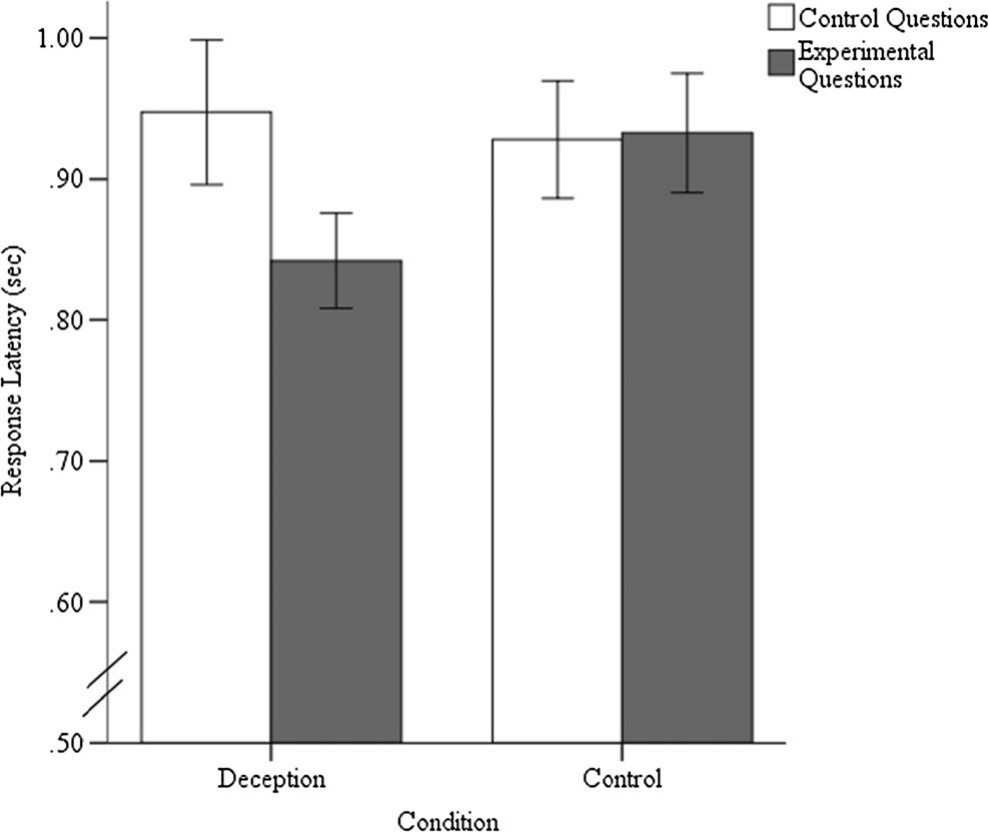
Response latencies across different conditions and question types. Between-subjects error bars represent ±1 *SE*

We conducted a repeated measures analysis of variance, with condition as the between-subjects variable (control vs. deception), questions type (control vs. experimental) and question number (eight questions per type) as within-subjects variables, and response latency as the dependent measure. Neither the main effect of question type (*p* = .052) nor that of deception condition (*p* = .525) was significant. However, the Question Type × Condition interaction was significant, *F*(1, 37) = 4.83, *p* = .034, *η*_p_^2^ = .12; see Fig. 2. To assess whether the difference between the control and experimental questions could be a cue to deception, we conducted paired-samples *t* tests for the deceptive and nondeceptive groups, with question type as the within-subjects variable and mean latency as the dependent measure. For the deception condition, we found that the mean response latency scores were significantly longer for the control questions, *M* = 0.95, *SD* = 0.23, than for the experimental questions, *M* = 0.84, *SD* = 0.16, *t*(20) = 2.82, *p* = .011, *d* = 0.61; see Fig. 2. For the control condition, the mean response latency scores were similar for control questions, *M* = 0.93, *SD* = 0.18, and experimental questions, *M* = 0.93, *SD* = 0.18, *p* = .89.

For completeness, we directly compared the response latencies to the experiment and control questions between the deception and nondeception groups. Experimental question response latencies in the deception group were not significantly different from the control question response latencies in the nondeception group, *t*(37) = –1.62, *p* = .11, nor were the experimental question response latencies in the deception group significantly different from those latencies in the nondeception group, *t*(37) = –1.69, *p* = .099. This lack of differences was likely due to power, because the significant difference between the experimental and control questions in the deceptive group was a within-subjects comparison, whereas these *t* tests were performed between subjects. However, the within-subjects comparison was what could be used as a cue to deception in applied settings, not the group-level comparison. Therefore, the lack of significance at the group level was of less interest.

## Discussion

The results were opposite to those in the previous literature: Liars showed shorter latencies when lying. Interestingly, liars did not respond faster than truth tellers overall. Liars only responded more quickly *when they were lying*, whereas truth tellers responded equally quickly to both groups of questions. These results suggest that in this ecologically valid scenario, liars answer more quickly when lying, not more slowly, as has previously been thought. Additionally, because no difference was found between the latencies of the experimental and control questions in truth tellers, we can be reasonably sure that these results are not merely a consequence of the difference in content between the experimental and control questions.

These findings may mean that in an airport setting, the cognitive-load aspect of lying is not as prominent as it is in laboratory situations. Because the “right answer” is known, the cognitive load induced by passing through a security check is likely lower for both truth tellers and liars than is answering the types of questions used in laboratory settings. The added cognitive load of lying is also reduced, because there is no need to actively create a new answer. This overall reduction in cognitive load, plus the reduction in load added by lying, likely means that the difference between truth tellers and liars in cognitive load is close to eliminated. However, this only explains why liars are not slower than truth tellers, not why liars are *faster* when lying

It could be that this reduction in response latencies is an effect of arousal or fear. In a more ecologically valid situation in which they are being questioned by an imposing figure, participants might experience more arousal or fear. Greater arousal is associated with faster response times in non-lie-detection fields of psychology (Eason & Harter, 1969), although this has not been consistently agreed upon within lie detection (Sporer & Schwandt, 2006). However, if individuals answer questions more quickly when aroused, then one would expect that participants would have answered all of the questions more quickly, not only the questions on which they were being deceptive. However, liars only responded more quickly when lying, so it is unlikely that the faster responses were due to increased arousal.

Alternatively, liars’ faster latencies may be a result of them either consciously or subconsciously responding more quickly when lying, to appear more confident in an attempt to deceive the officer. The main point supporting this interpretation is the finding that liars are only faster when lying. If liars were trying to be as fast as possible in order to appear more confident when engaging in deception, they would likely do so only for those questions. This decrease in response latencies could possibly also be an unconscious strategy. The response latencies were all under 1 s, and were even lower when participants engaged in deception. Therefore, it is unlikely that participants had time to deliberately contemplate answering more quickly to only the questions they answered deceptively, while answering less quickly to questions they answered truthfully. One possibility is that hearing a question in which one must answer deceptively triggers an impulse to respond as quickly as possible, especially for yes/no questions that the liar is anticipating. Future research should differentiate between the conscious and unconscious mechanisms. Following increased investigation, these findings may show a potentially powerful tool in deception detection.

Interestingly, the majority of the lie detection literature has shown longer response latencies, but only when lying about arbitrary stimuli and using open-ended responses. These results highlight the notion that certain cues to deception do not provide a “one size fits all” solution. The context and types of questions matter in terms of how people engage in deception. Perhaps these differences as a consequence of context and question type are some of the reasons why the research on deception detection has been so divided. Likely, reliable cues to deception exist, but different cues are used that depend on the task and environment. Thus, for research on deception to be useful, it should focus on investigating cues to deception in the explicit scenario and on the types of questions in the environment of interest.

A second area of future research should be expansion of the use of VR in lie detection research. Our findings that participants experienced high immersion and that truth tellers and liars did so equally mean that VR may be a solution to the problem of ecological validity in lie detection research. This problem is longstanding and well-known, but so far there has been little progress on a solution (see, e.g., DePaulo et al., 2003). VR would be a way for researchers to have the realistic environments needed, as well as having the experimental control that laboratory studies offer. In fact, VR allows researchers to have even more control than in most laboratory studies, because VR allows them to fully control each movement and word by the interviewer, which is an impossibility for interviewers in the real world. Using VR would also substantially reduce the cost of setting up realistic environments and realistic action tasks. Although VR might solve part of the ecological validity problem, it cannot address the lack-of-motivation problem. In real life, someone trying to smuggle illegal items through a security checkpoint faces arrest, a prison sentence, and possibly a violent confrontation. VR cannot replicate these very real dangers, and if it could, it would not be ethical to use it. However, VR may be the closest that we can get to replicating these situations.

Our research has uncovered a new, potentially useful cue to deception. Additionally, our results call into question the generalizability of previous research on response latencies as a cue to deception. They highlight the importance of ecological validity when researching lie detection, and uncover a new potential tool for enhancing lie detection in real-world scenarios.
